# Enhanced Photocatalytic Performance of TiO_2_@Er-Hydroxyapatite Composite for Cationic Dye and Drug Removal

**DOI:** 10.1021/acsomega.4c06314

**Published:** 2025-02-03

**Authors:** Rafael Lisandro P. Rocha, Alan Ícaro S. Morais, Francisca P. Araujo, Luzia Maria C. Honório, Marcos P. Silva, Marcelo B. Furtini, Ewerton G. Vieira, Edson C. da Silva-Filho, Josy A. Osajima

**Affiliations:** † Interdisciplinary Advanced Materials Laboratory (LIMAV), Materials Science and Engineering Graduate Program, Federal University of Piaui (UFPI), Teresina, PI 64049-550, Brazil; ‡ Department of Chemistry and Physics - Center for Agrarian Sciences, UFPB, Areia, PB 58051-900, Brazil; § Instituto Federal do Maranhão - Campus Buriticupu, IFMA, Buriticupu, MA 65393-000, Brazil

## Abstract

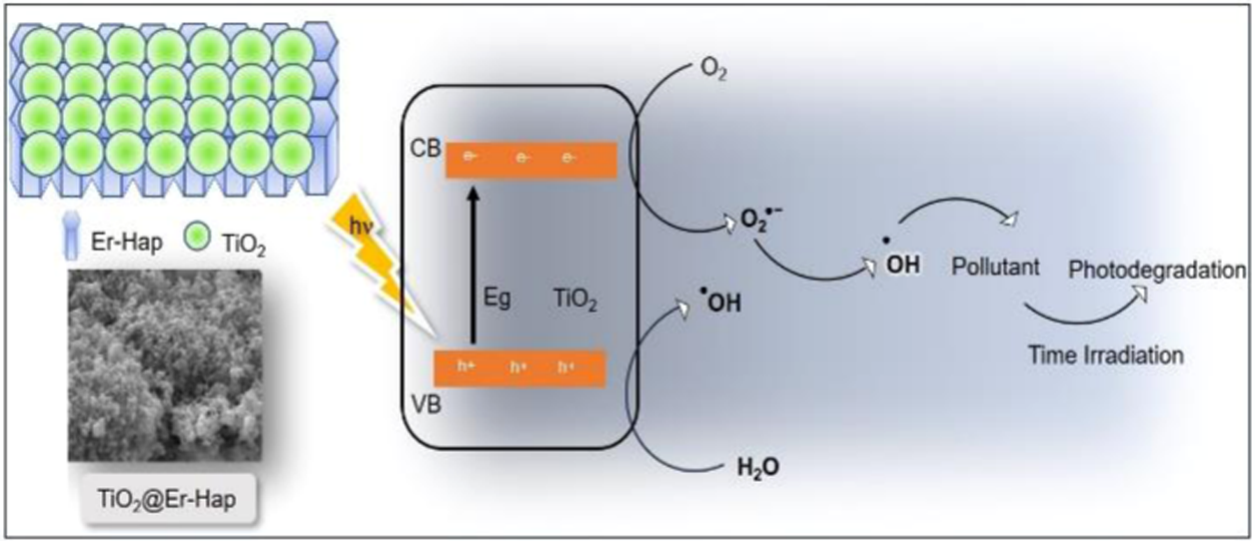

Photocatalysis heterogeneous is an essential method for water treatment. In this study, a novel photocatalytic erbium-doped hydroxyapatite-decorated titanium dioxide (TiO_2_@Er-Hap) was obtained using the sol–gel method and applied to remove ciprofloxacin (CIP) drug and methylene blue (MB) dye contaminants. The sample’s structural, physical, morphological, and photocatalytic properties were investigated. X-ray diffraction (XRD) confirmed the presence of the anatase phase in TiO_2_@Er-Hap. The oxide nanoparticles were deposited on the Hap surface, and the proposed material presented a band gap energy of 3.33 eV. Furthermore, TiO_2_@Er-Hap presented a larger surface area when compared with the material not decorated with the oxide. Photocatalysis tests performed under ultraviolet (UV) irradiation showed that TiO_2_@Er-Hap removed MB dye (100%) and CIP (71.16%), despite low mineralization evidenced in TOC analysis. The irradiated pollutant solution after the photocatalysis process was nontoxic, as observed in the ecotoxic test, indicating that the photocatalytic worked. Inhibitor tests showed that the ^•^OH radicals were the main species involved in the tests performed. The material demonstrated activity even after five consecutive cycles of use. Finally, the results suggest that TiO_2_@Er-Hap is a suitable candidate for water decontamination via photocatalytic processes.

## Introduction

Population growth has brought numerous advances in industrial and commercial activities. However, despite being essential for human life, the intensification of the urbanization process attenuated the level of contaminants and environmental disorders, increasing the presence of contaminants in the water, such as dyes, heavy metals, pesticides, and pharmaceuticals, harming the ecosystem and it causes severe health damage.^1−3^ In particular, organic pollutants represent a serious concern due to their toxic nature and also because they do not respond well to conventional treatments.^4^ Different treatment methodologies are used according to their economic, effective, and environmentally correct properties to minimize the impacts generated, for example, electrochemical treatments, ozonation processes, flotation, adsorption, and photocatalytic processes.^5,6^ Heterogeneous photocatalysis is an efficient technique for removing organic pollutants in wastewater. It works under natural pressure and ambient reaction conditions, and the material used as a catalyst can be separated after the degradation process.^7^ Photocatalysis has been considered a green strategy to produce oxygenated radicals, especially the ^•^OH radical, which are useful for oxidizing and degrading the structure of contaminants.^8^

Hydroxyapatite (Hap) is a suitable candidate for use in environmental remediation. Hap, with a structural formula (Ca_10_(PO_4_)_6_(OH)_2_), is an inorganic compound made up of calcium and phosphate minerals with a Ca/P molar ratio close to 1.67 (stoichiometric ratio). With an emphasis on environmental remediation, Hap has been considered a promising compound for environmental remediation, and this property is adjusted with its adsorption capacity.^9^ Moreover, it allows the replacement of ions and, consequently, structural changes, depending on the fraction and size of the cations and/or anions, to be replaced, thus making it a multifunctional material in environmental mitigation.^3,10^ Strategies such as the functionalization of the structure of this phosphate, such as doping, impregnation, and heterojunction, among others, can provide a material for photocatalytic application.^3,9,11,12^

The immobilization of semiconductors on the surface of Hap is also an exciting proposal for Hap in environmental remediation. It is well-known that classical semiconductor materials such as ZnO and TiO_2_ have good physicochemical characteristics for removing pollutants via heterogeneous photocatalysis.^13,14^ However, the surface area, ultraviolet (UV) photoactivation, and recombination of photogenerated charge carriers are recognized as the main limitations of these oxides. The literature reports that immobilizing TiO_2_ on cosorbents and/or catalytic supports is an excellent way to achieve effective materials for removing pollutants.^8,14,15^ The literature reported that TiO_2_/Hap presented stability in the methyl orange degradation.^16−17,18^ Furthermore, a photocatalytic potential in nanostructures of TiO_2_@Hap against the degradation of rhodamine-B in an aqueous solution under UV irradiation was also related.^19^ Go et al.^20^ recently reported multivariable Hap terms when evaluating the degradation of the antibiotic levofloxacin under UV radiation, with the response surface methodology used to model and design operational parameters to obtain optimized experimental conditions.

Some studies have reported the introduction of transition metals such as Ti^4+^,^21^ Fe^3+^,^22^ Zn^2+^,^23^ and Cu^2+^^24^ in the Hap matrix for contaminant removal via photocatalysis. In general, doping can alter electronic levels and shift the absorption edge, with an improved response due to the emergence of new energy levels.^25^ Rare earth (RE) metal ions are excellent candidates for replacement.^26^ Although Hap materials doped with RE are widely explored in the biomedical field, it is known that introducing these ions into a host lattice can modify the general and optical properties of the doped materials to be obtained.^27,28^ Jimenez-Flores et al.^29^ reported the obtention of Hap doped with terbium metal and showed that doping improved the textural and luminescent properties of calcium phosphate, resulting in satisfactory photocatalytic response for organic pollutant removal.^29^

Recently, using Hap doped with iron and coated with TiO_2_ proved efficient in pollutant degradation.^30^ It is known that doping the Hap network with metals can favor the increase of the surface area,^31^ which is a parameter of interest when the application depends on the surface properties as is the case of heterogeneous photocatalysis—considering that Hap is not an efficient photocatalyst. The use of Er-Hap as support for TiO_2_ nanoparticles aims to create a material with enhanced photocatalytic properties in this study. This work proposes a study of the structural, physical, and morphological properties of Er-Hap and Er-Hap decorated with TiO_2_ (named as TiO_2_@Er-Hap). The material doped and supported with photocatalytic oxide was investigated to remove organic pollutants such as the cationic methylene blue dye and ciprofloxacin drug via photocatalysis.

## Experimental Section

### Chemicals

The reagents used were calcium hydroxide (Vetec, Ca(OH)_2_), dibasic ammonium phosphate (Neon, (NH_4_)_2_HPO_4_), erbium(III) nitrate pentahydrate (Sigma-Aldrich, Er(NO_3_)_3_·5H_2_O), ethyl alcohol 99.8% (Dinâmica), titanium isopropoxide 97% (Sigma-Aldrich), methylene blue 97% (Dinâmica), and ultrapure water. They are used without purification. The amounts were stoichiometrically determined according to a Ca/P molar ratio of ∼1.67.

### Synthesis of Er-Hap

Er-Hap was synthesized using the suspension–precipitation method^32^ with Ca(OH)_2_, (NH_4_)_2_HPO_4_, and Er(NO_3_)_3_·5H_2_O as the precursor reagents. The addition of the erbium doping (*x*_mols/Er_ = 2.50% or 0.025 mol) occurred in proportion to the percentage of calcium hydroxyapatite (Ca_10–*x*_Er_*x*_(PO_4_)_6_(OH)_2_). The reagents Ca(OH)_2_ and (NH_4_)_2_HPO_4_ were dissolved in 100 mL of ultrapure water. It was left stirring for 3 h at pH = 11 and 25 °C. The resulting colloidal dispersions were centrifuged and washed with ultrapure water, and the formed product was dried at 110 °C for 12 h. Finally, the obtained powder was deagglomerated with a mortar and pestle and sieved with an aperture of 425 μm.

### Synthesis of Er-Hap Decorated with TiO_2_

TiO_2_@Er-Hap photocatalytic was synthesized by the sol–gel method, as suggested in the literature.^6^ For this, 1 g of Er-Hap was stirred in 100 mL of ethyl alcohol for 30 min. Then, 6.0 mL of titanium isopropoxide was slowly added to the Er-Hap solution, and the mixture was left stirring for another 30 min. After this stirring, 6.0 mL of ultrapure water was slowly added, and the mixture was left stirring for another 30 min. The solution was allowed to stand for 24 h and then dried in an oven at 75 °C. Finally, the material was calcined at 400 °C for 2 h with a heating rate of 10 °C/min. The final material was named TiO_2_@Er-Hap.

### Characterization

The microstructural analysis of the samples was carried out using X-ray diffraction in Labx-XRD 600 equipment from Shimadzu, with Cu Kα radiation (λ = 1.5406 Å) of 2θ in the range between 2 and 75°, with a sweep rate of 2°/min. Infrared spectra were collected using the 1% KBr (potassium bromide) pellet method in an Agilent Cary 660 Fourier transform infrared spectroscopy (FTIR) equipment in the 4000–400 cm^–1^ region with 120 scans and a resolution of 4 cm^–1^. Scanning electron microscopy and energy dispersive spectroscopy (SEM-EDS) using field emission electron source equipment performed the morphological characterization of the surface and the pore structure of the samples (FEI, model Quanta FEG-250). The samples were mounted on “stubs” using double-sided carbon tape and covered in gold. SEM conditions were 10KV, and the working distance was 10 mm with point 3. Surface properties were performed on a Quantachrome-NOVA instruments surface area analyzer. The surface area was determined by the multipoint BET (Brunauer–Emmett–Teller) method using the data as a function of the relative pressure (*P*/*P*_0_) of the nitrogen at 77K. Before measurements, the samples were degassed at 100 °C for 24 h.

The material’s band gap (Eg) was performed using a Shimadzu Model UV-3600 spectrophotometer with diffuse reflectance accessory monitoring the region from 200 to 800 nm. It was calculated through a series of mathematical transformations proposed in the Kubelka–Munk method.^33^ Photoluminescence (PL) spectra were obtained using a Horiba-JobinYvon Fluorolog-3.

### Photocatalytic Investigation

The photocatalytic capacity of the TiO_2_@Er-Hap material was investigated by using methylene blue dye (MB), a typical model pollutant from photocatalytic tests, and a ciprofloxacin (CIP) drug. The dye concentration used was 10 ppm, while the drug concentration was 6 ppm. The photodegradation was carried out by a radiation box containing a 150 mL borosilicate reactor. The temperature was controlled at 25 °C using a thermostat bath. The UV irradiation source was a 160 W mercury vapor lamp without a bulb. The amount of photocatalyst was 0.5 g·L^–1^. Before irradiation, the MB solution with the photocatalyst was stirred in the dark for 60 min to achieve adsorption–desorption equilibrium. Changes in pollutant concentration were monitored using a UV–vis spectrophotometer (Agilent Technologies Cary 60) by the absorption band at 664 nm for MB and 274 nm for CIP. The degradation rate in each case was calculated by eq 1:^34^
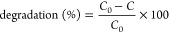
1where *C*_0_ and *C* correspond to the absorbances of the pollutant solution before and after irradiation, respectively.

The rate constant in heterogeneous photocatalysis mediated by the catalyst synthesized used the first-order kinetic models, described as shown in eq 2:^35^
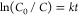
2where *C*_0_ and *C* correspond to the initial absorbance of MB and the absorbance of the dye at time *t*, respectively; *k* is the apparent rate constant (min^–1^), and *t* is the time (min).

The second-order kinetic models are mathematically described using eq 3:^35^
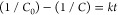
3where C_0_ and C correspond to the initial absorbance of MB and the absorbance of the dye at time *t*, respectively; *k* is the apparent rate constant (mol^–1^L min^–1^), and t is the time (min).

The suppressor test was performed to determine the species involved in the photocatalytic activity of the materials. For this, silver nitrate (AgNO_3_), tetrasodium ethylenediaminetetraacetate dihydrate (EDTA), and methanol (MeOH) were used as scavengers of electrons (e^–^), holes (h^+^), and hydroxyl radicals (^•^OH), respectively.^6,36,37^ Reuse tests also were performed, where at the end of the photocatalytic process, the material was washed three times with deionized water and dried for 24 h in an oven at 100 °C. After this time, the material was weighed again following the same photocatalytic procedure to determine the percentage of discoloration of the MB dye, estimated by eq 1.

The irradiated solutions were analyzed for mineralization using total organic carbon (TOC) analysis using a GE Sieviers Innovox analyzer. The TOC determination was made as suggested in the literature^38^ from to mixture of the diluted volume of the irradiated solution with H_3_PO_4_ (6.0 mol·L^–1^) and Na_2_S_2_O_8_ (30% m/v) as acidifying and oxidizing solutions, respectively.

### Ecotoxicity

The toxicity of irradiated pollutant solutions was investigated by using the bioassay with *Artemia saline*. For this, microcrustaceans were cultivated for 48 h in a synthetic saline solution under lighting and oxygenation. After this time, ten nauplii were added to a mixture of the saline solution and the irradiated photocatalysis test solution (1:1 v/v). The number of living nauplii was determined after 24 and 48 h.

## Results and Discussion

### Structural Characterization

**Figure 1:**
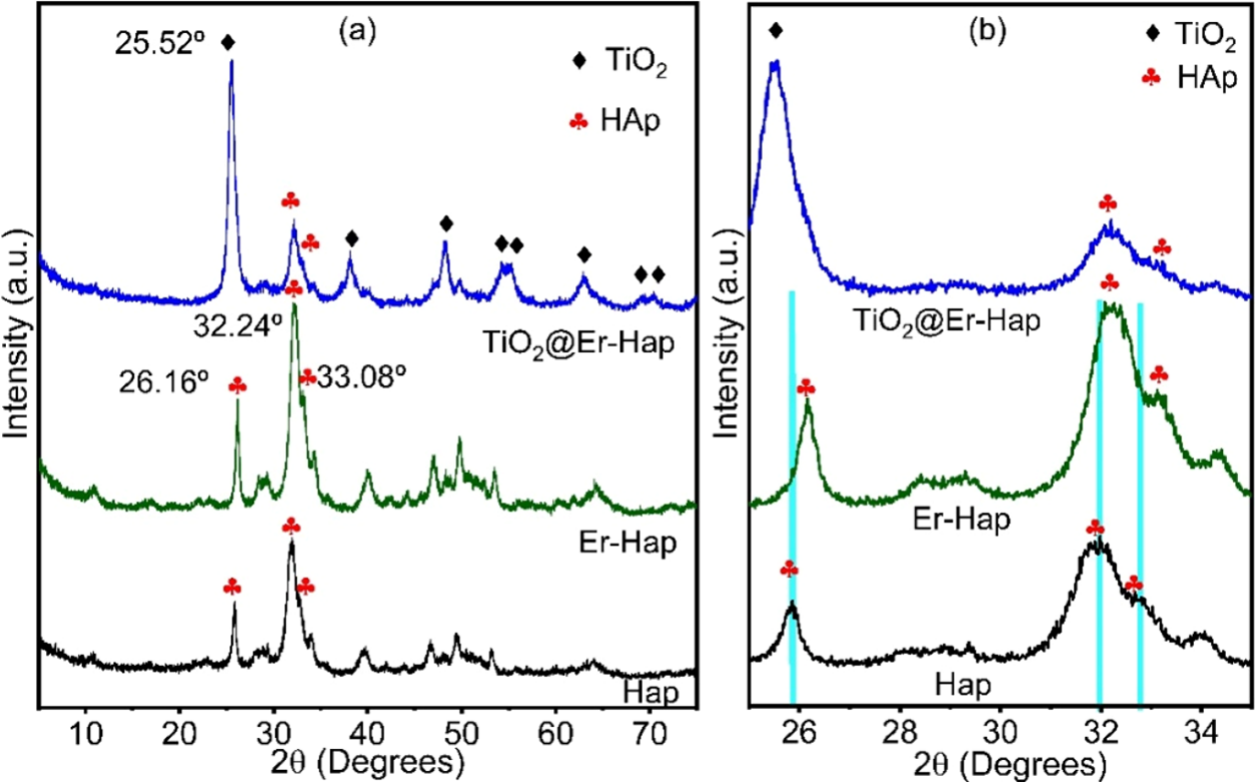
(a) X-ray diffractogram of the samples based on Hap and (b) magnification to visualize the displacement of peaks in the samples.

The structural analysis of the materials obtained was evaluated based on the XRD diffractograms shown in Figure 1. In the Er-Hap diffractogram, characteristic peaks of crystalline Hap were identified in the region of 2θ = 26.16, 32.24, and 33.08°, which are attributed, respectively, to planes (002), (211), and (300), considering JCPDS Card number: 00-003-0747.^32^ Analyzing the diffractograms more carefully in the region between 2θ = 25 and 35°, it was evident that the original Er-Hap peaks were shifted larger toward the side with the higher diffraction angle, as shown in Figure 1b. The Ca^2+^ ion (0.099 nm) has a smaller radius than the Er^3+^ ions (0.088 nm), and the replacement of divalent ions from the host lattice by dopant ions promotes these changes in the diffractogram profile, as reported previously.^39,40^ Thus, our results suggest that Er^3+^ replaced Ca^2+^ ions. This behavior is acceptable because the dopant concentration used in Er-Hap in the synthesis process is low. Analyzing TiO_2_@Er-Hap diffractogram and comparing with JCPDS card 01-084-1286 for TiO_2_ were identified typical peaks of TiO_2_ in the anatase phase at 2θ = 25.52, 38.16, 48.26, 54.22, 55.26, 62.93, 69.03, 70.04, and 75.31° indexed to (101) (004) (200) (105) (211) (204) (116) (220), and (215) planes, respectively.^41^ These results show that TiO_2_ was successfully obtained in the investigated composite. In addition, the peaks associated with Hap were identified in the diffractogram of this sample.

**Figure 2:**
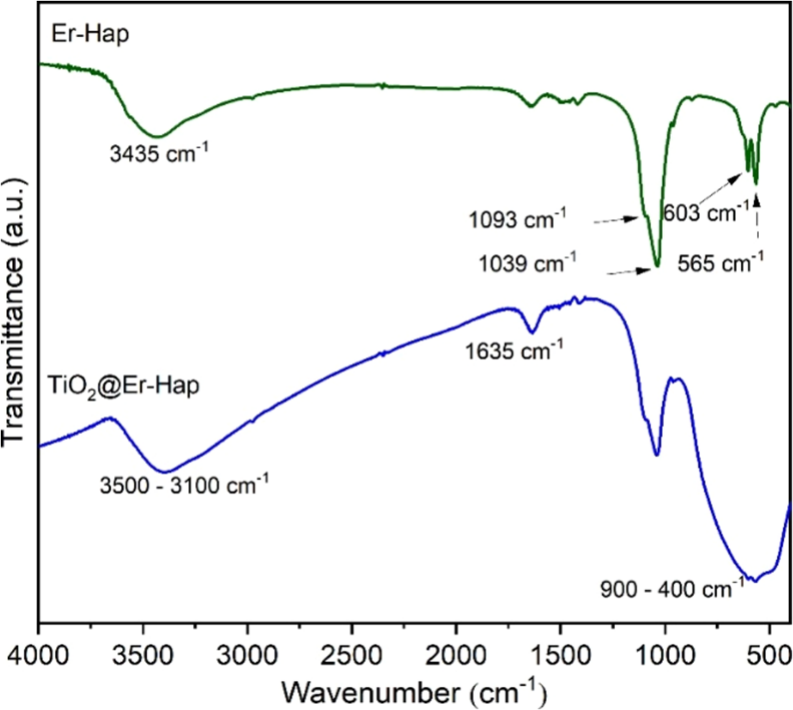
Spectroscopic characterization using FTIR analysis on the Er-Hap and Er-Hap@TiO_2_ samples.

Infrared spectra of Er-Hap and TiO_2_@Er-Hap are shown in Figure 2. In the Er-Hap sample, observing characteristic bands of the Hap ceramic was possible. For example, the band at 3435 cm^–1^ corresponds to the stretching of the O–H bond in hydroxyl groups. The bands around 1093 and 1039 cm^–1^ are attributed to asymmetric deformation in phosphate groups (PO_4_^–3^), while the bands at 603 and 565 cm^–1^ are due to the deformation of the phosphate groups.^42^ For the TiO_2_@Er-Hap sample, the band noted between 3500 and 3100 cm^–1^ is related to the stretching binding vibration of the hydroxyl groups. The band at 1635 cm^–1^ occurs due to the deformation vibrations of the hydroxyl group sample. The band noted between 3500 and 3100 cm^–1^ is related to the stretching binding vibration of the hydroxyl groups, and the bands at 1635 cm^–1^ occur due to the deformation vibrations of the hydroxyl group. The broadbands between 900 and 400 cm^–1^ are the characteristic bands of TiO_2_ in its anatase phase,^43^ indicating the presence of the TiO_2_ oxide. Bands identified at 603 and 565 cm^–1^ in the spectrum of the Er-Hap sample were also present in the spectrum of the material decorated with TiO_2_ even after the heat treatment process was carried out.

### Morphological Analysis and Elemental Composition

**Figure 3:**
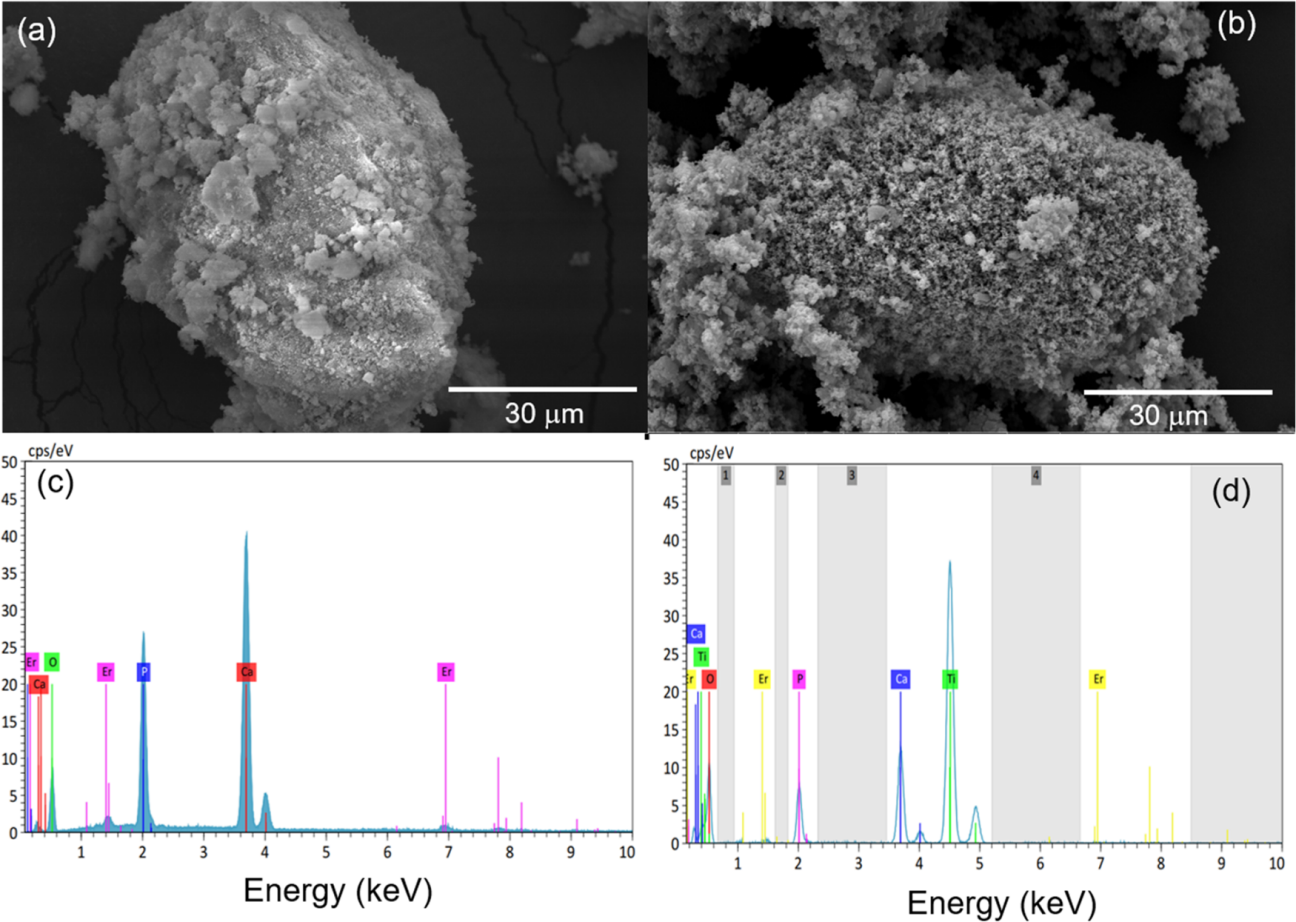
Morphological analysis was performed with SEM technique for (a) Er-Hap and (b) TiO_2_@Er-Hap and mapping the chemical composition using EDS analysis in (c) Er-Hap and (d) TiO_2_@Er-Hap materials.

The morphology of hydroxyapatite-based materials obtained was investigated by using SEM analysis, and images are shown in Figure 3. In Er-Hap material, the presence of irregular grain morphology was observed, as demonstrated in Figure 3a. For the TiO_2_@Er-Hap sample, SEM images in Figure 3b showed the presence of the spherical TiO_2_ nanoparticles covering the surface of the doped Hap, indicating that the oxide decorated the surface of the Hap support. The chemical composition of the obtained samples was examined using EDS analysis, and the results are presented in Figure 3c,d. Regarding these results, peaks associated with Ca and P constituents of the Hap structure were identified. The Er peak observed indicates the metal incorporated as a dopant in the calcium phosphate structure in both samples. In addition, the Ti peak found in the TiO_2_@Er-Hap sample is associated with the presence of the oxide that decorates the surface of the Hap.

### Gap Energy and Textural Properties

The band gap values of the samples were estimated from the Kubelka–Munk function and Tauc’s relation. The data was 4.89 and 3.33 eV for Er-Hap and TiO_2_@Er-Hap, respectively. It is known that the band gap of Hap displays a value of 5.2 eV.^44^ Our results demonstrate that doping in the Hap host lattice is beneficial in promoting the lowering of this phosphate’s band gap energy value. The decrease in the Eg value observed in Er-Hap indicates that the Ca^2+^ sites were replaced by Er^3+^, creating defect states that promote a decrease in this parameter. The nature of the defects generated can vary according to the RE ion used to dope the Hap structure.^26^ However, there is a consensus that oxygen vacancies are the main types of defects related to the decrease in band gap energy of Hap when doped with RE metal ions.^26,45^ A decrease from Eg value to Hap doped with other RE metal ions such as Tb^3+^,^46^ Ce^4+^,^47^ and Pr^3+^^45^ has been described in the literature. About TiO_2_, the band gap energy of TiO_2_ anatase is reported as being ∼3.2 eV.^48^ In the TiO_2_@Er-Hap sample, the calculated Eg value was 3.33 eV, representing a slight increase compared to the value commonly declared for TiO_2_. The band gap energy is an important parameter in studies involving materials for photocatalytic applications because the radiation can be decisive in heterogeneous photocatalysis. In addition to the band gap energy, the surface area can affect the photocatalytic response, as degradation reactions occur at the solid–liquid interface. Here, the BET method was used to estimate the surface area from nitrogen adsorption–desorption. It was found that the surface area of the Er-Hap was 71.32 m^2^g^–1^, while the TiO_2_@Er-Hap presented a value of 93.67 m^2^g^–1^. Covering Hap with oxide favored increased surface area in the TiO_2_@Er-Hap sample. The immobilization of oxides on surfaces with such properties has been presented as an efficient strategy to obtain desirable materials for applications whose material performance depends on this parameter.^36,49^

### PL Analyses

PL spectra for TiO_2_@Er-Hap were collected in this study using an excitation source at 364 nm, whose profile is presented in Figure S1. There was a strong emission at ∼448 nm and other emissions of discrete intensity at 506 and 532 nm. For anatase TiO_2_, bands that appear in the spectra are due to self-trapped excitons, surface states, quantum confinement, and oxygen vacancies.^50^ Bands centered between 490 and 540 nm are expected in the emission spectrum of the anatase phase of TiO_2_ and are associated with oxygen vacancies,^51^ considered charge carrier traps (e^–^/h^+^), making their recombination difficult.

### Photocatalytic Efficiency of TiO_2_@Er-Hap

**Figure 4:**
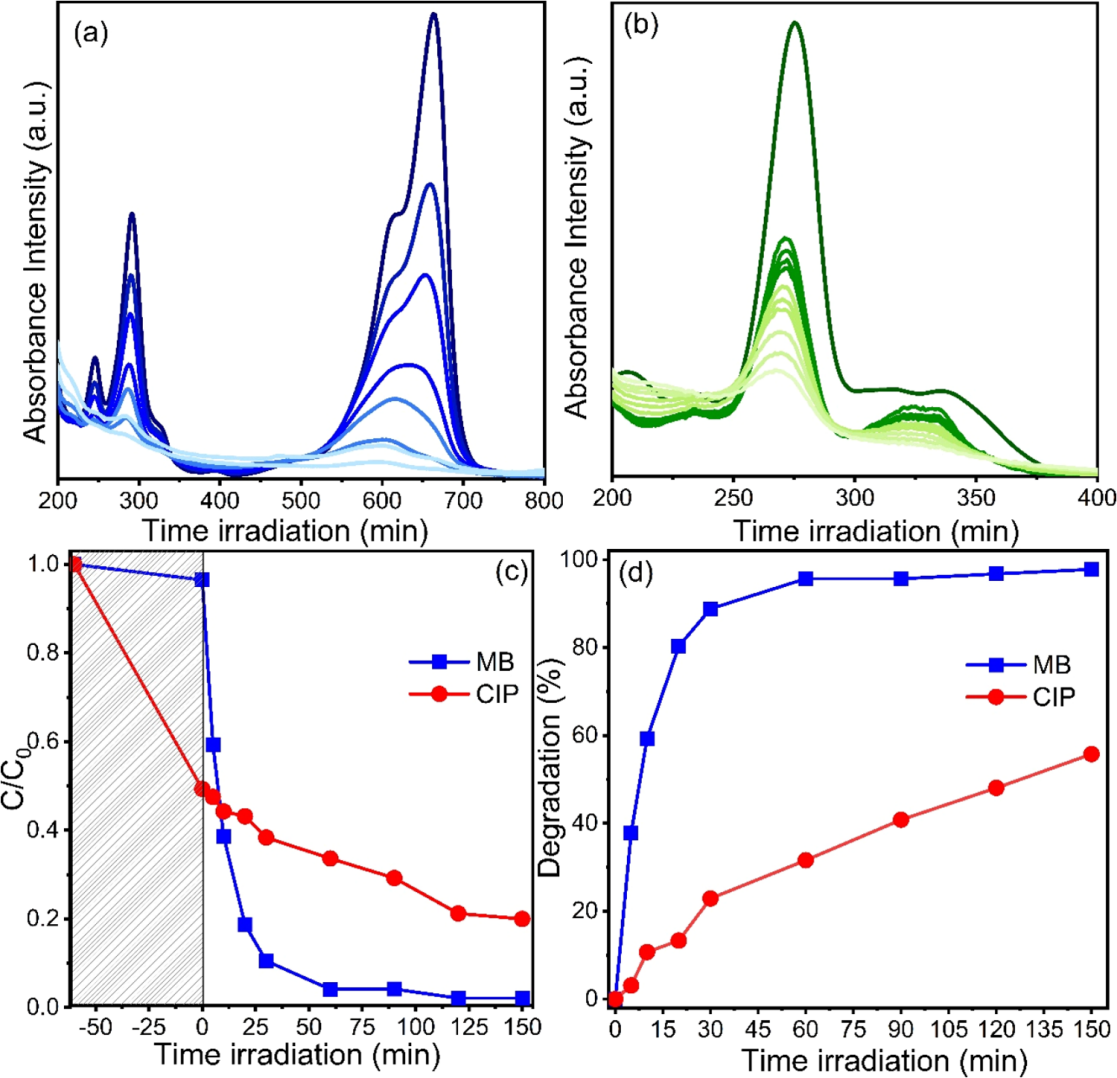
Spectral variation in photocatalytic tests performed with TiO_2_@Er-Hap in (a) MB dye and (b) ciprofloxacin drug; (c) *C*/*C*_0_ ratio and (d) rate degradation of the pollutants.

The photocatalytic efficiency of the TiO_2_@Er-Hap material was investigated using the MB dye and CIP drug as pollutants under UV irradiation conditions. The results obtained in the photocatalytic tests are shown in terms of the spectral variations and from the quantitative interpretation of these results, as seen in Figure 4. In systems containing TiO_2_@Er-Hap as a photocatalyst, when the irradiation progressed, the dye band at 664 nm, typical in MB spectra, progressively decreased, and after 150 min, it was almost nonexistent. It was observed that the 277 nm band also progressively decreased as the system was irradiated by using the photocatalyst in the drug solution, suggesting that the catalyst effectively removed the CIP pollutant. Under a quantitative approach and considering the *C*/*C*_0_ ratio as a function of irradiation time (Figure 4c,d), it was noted that this parameter decreased over the irradiation time in both systems containing the dye or drug as a pollutant. This result indicates that the concentration of substances decreases within each system due to degradation promoted by the catalyst. However, TiO_2_@Er-Hap demonstrated greater affinity with the pollutant CIP because the *C*/*C*_0_ ratio decreased by ∼51.0% from the initial value in dark adsorption, as seen in Figure 4c. The experimental results calculated MB and CIP degradation rates for the investigated systems with observed values of 97.80 and 55.77%, respectively, as shown in Figure 4d.

Recent studies reported that the photocatalytic performance of pure TiO_2_ synthesized under similar conditions to oxide that decorated Er-Hap was 60% under UV irradiation.^41^ The photocatalytic degradation of CIP mediated by TiO_2_ was reported by Zhang et al. as 42.0%.^52^ It is evident that TiO_2_@Er-Hap material showed a greater capacity for removing the MB pollutant, suggesting that supporting TiO_2_ in Er-Hap can benefit the photocatalytic activity of this oxide. Based on the characterization results, the efficiency of TiO_2_@Er-Hap in removing the pollutants tested is associated with the largest surface area demonstrated for this material. The increase in surface area causes an increase in the availability of active sites for the dye–semiconductor interaction and, consequently, the photochemical reactions developed at this solid–liquid interface.^53^ In addition, it was also possible to improve the conduction efficiency of the electron/hole pair, favoring the advanced oxidative process in the formation of reactive oxygen species (ROS).^6^

**Figure 5:**
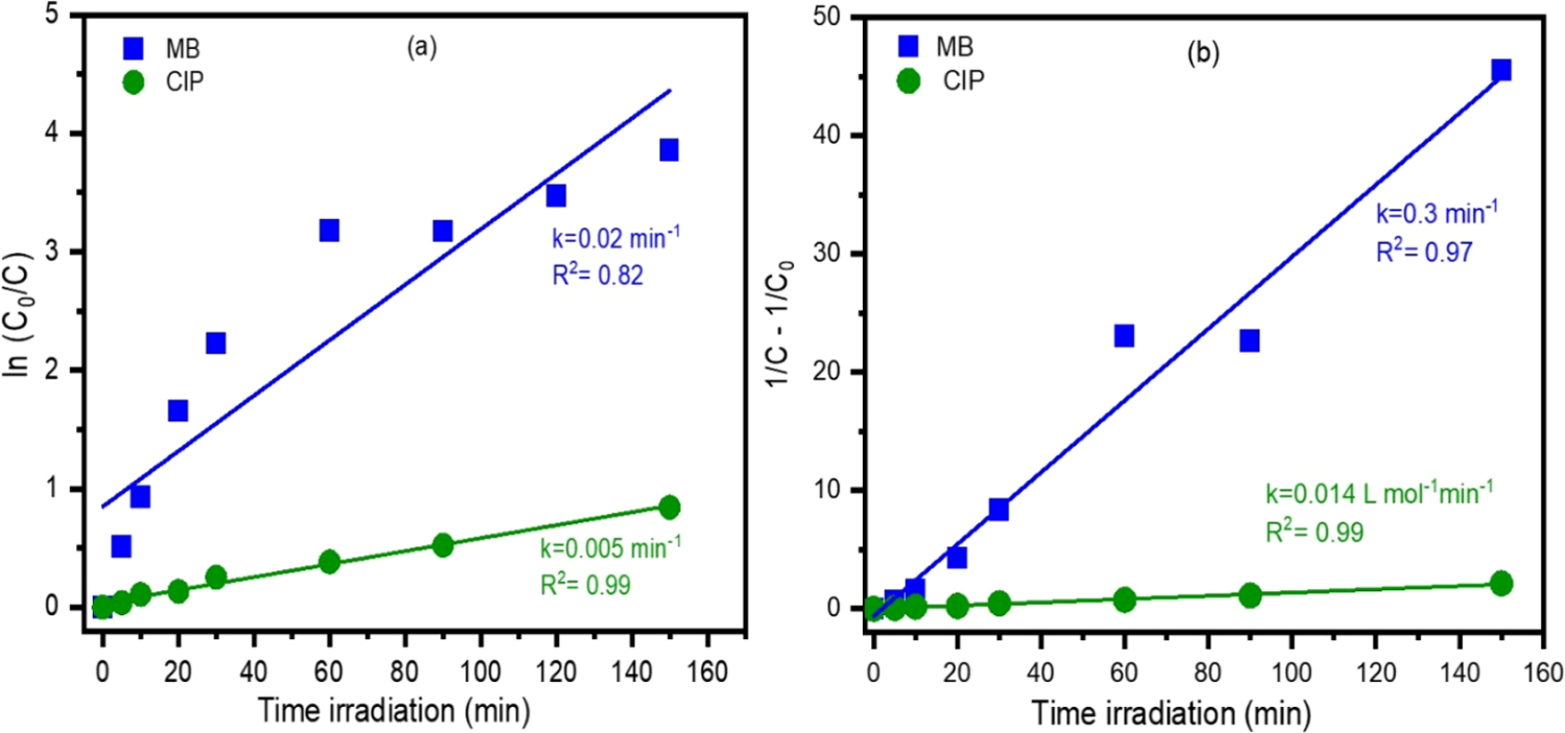
(a) First-order and (b) second-order kinetics with the rate constant for TiO_2_@Er-Hap in MB and CIP removal.

The photocatalysis data were fitted to first- and second-order mathematical models; the results are shown in Figure 5. In each case, the constant rate was determined from the slope of the line, and *R*^2^ data were calculated. The decomposition of MB by TiO_2_@Er-Hap presented a higher rate constant compared to conventional systems using CIP as a pollutant. Comparing the data with each other based on the *R*^2^ value, it was clear that the data fit better to the second-order model in systems containing MB. This result suggests that adsorption plays an important role in photocatalytic systems using TiO_2_@Er-Hap.^35^ Conversely, a photocatalytic study involving CIP degradation demonstrated that the data were adjusted satisfactorily to the first-order and second-order models, as determined through the *k* values.

### Dosage of Photocatalyst

**Figure 6:**
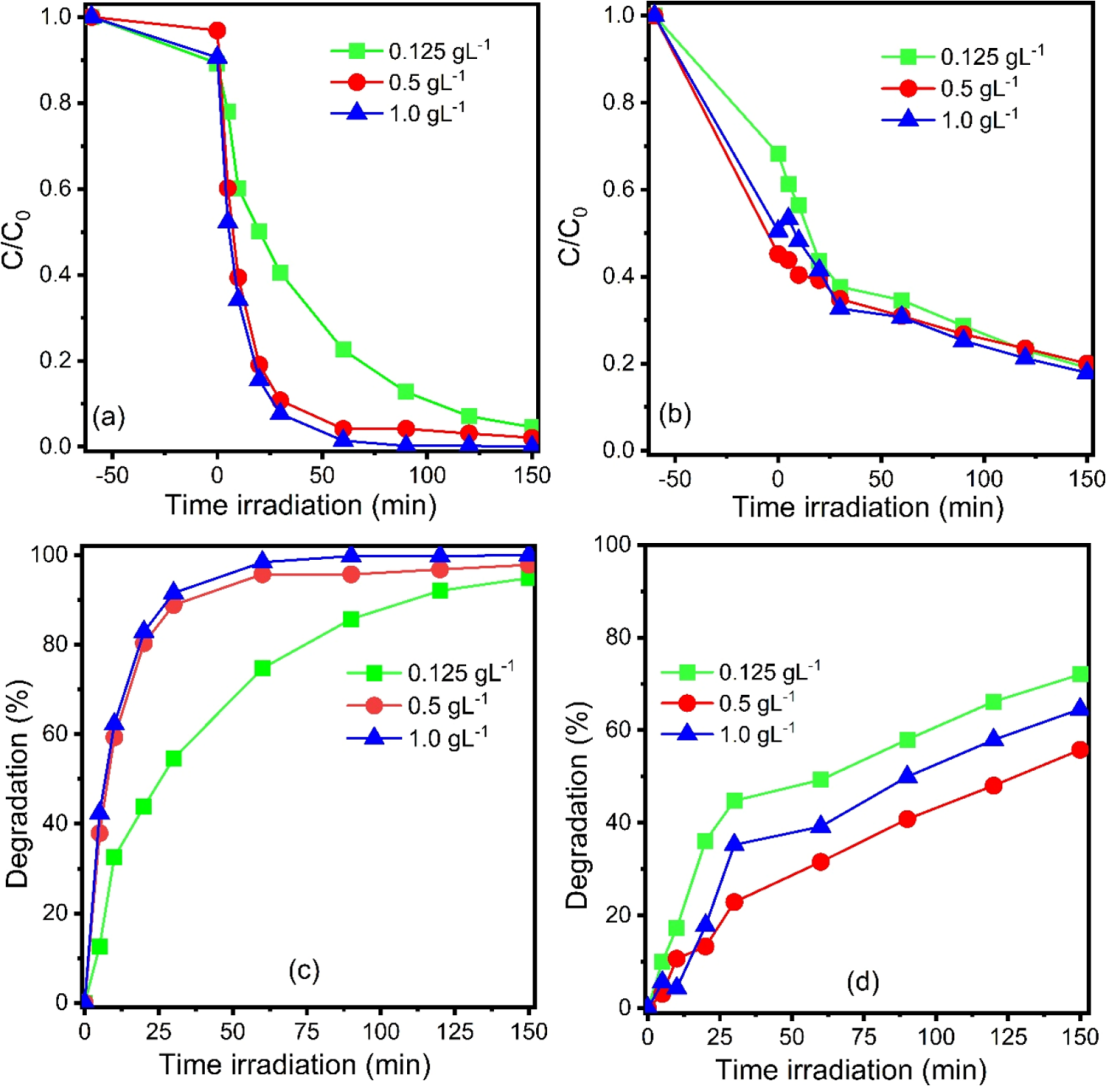
*C*/*C*_0_ versus irradiation time in systems performed with (a) MB and (b) CIP, and degradation rate obtained for photocatalytic systems containing (c) MB and (d) CIP pollutants.

In this work, we investigated the effect of the sample’s concentration on the degradation ability of MB and CIP, considering concentrations 0.125, 0.5, and 1.0 g·L^–1^ of the material. The results were expressed regarding the *C*/*C*_0_ ratio as a function of the irradiation time and rate degradation, as seen in Figure 6. In systems performed with MB, it was evident that with increasing concentration, the removal capacity of the pollutant also increased (Figure 6a). The MB degradation rate (Figure 6c) was 95.43, 97.80, and 100.0% in tests using a material with concentrations of 0.125, 0.5, and 1.0 g·L^–1^, respectively. When CIP was used as a pollutant, the *C*/*C*_0_ ratio decreased as a function of the irradiation time (Figure 6b). The degradation rate (Figure 6d) after 150 min of the irradiation was 71.16, 55.7, and 63.86% when catalyst concentrations were 0.125, 0.5, and 1.0 g·L^–1^, respectively.

In general, the increase in removal efficiency in the function of the dosage observed for MB is associated with an increase in solid–liquid interaction sites and the greater availability of charge carriers that will be responsible for the contaminant degradation reactions. Similar behavior was reported in other studies involving materials based on TiO_2_.^54^ For results obtained in photocatalytic systems performed with CIP as a pollutant, this behavior was not similar to that observed for the dye. The greatest removal capacity occurred when the photocatalyst was present at the minimum dosage of 0.125 g·L^–1^. It means that there is an optimal catalyst dosage for each system tested. In other words, the effect of catalyst amount on photocatalytic performance may differ depending on the material-pollutant interactions, as observed in the behavior profile during the dark adsorption of MB and CIP.

### Inhibitor Test

**Figure 7:**
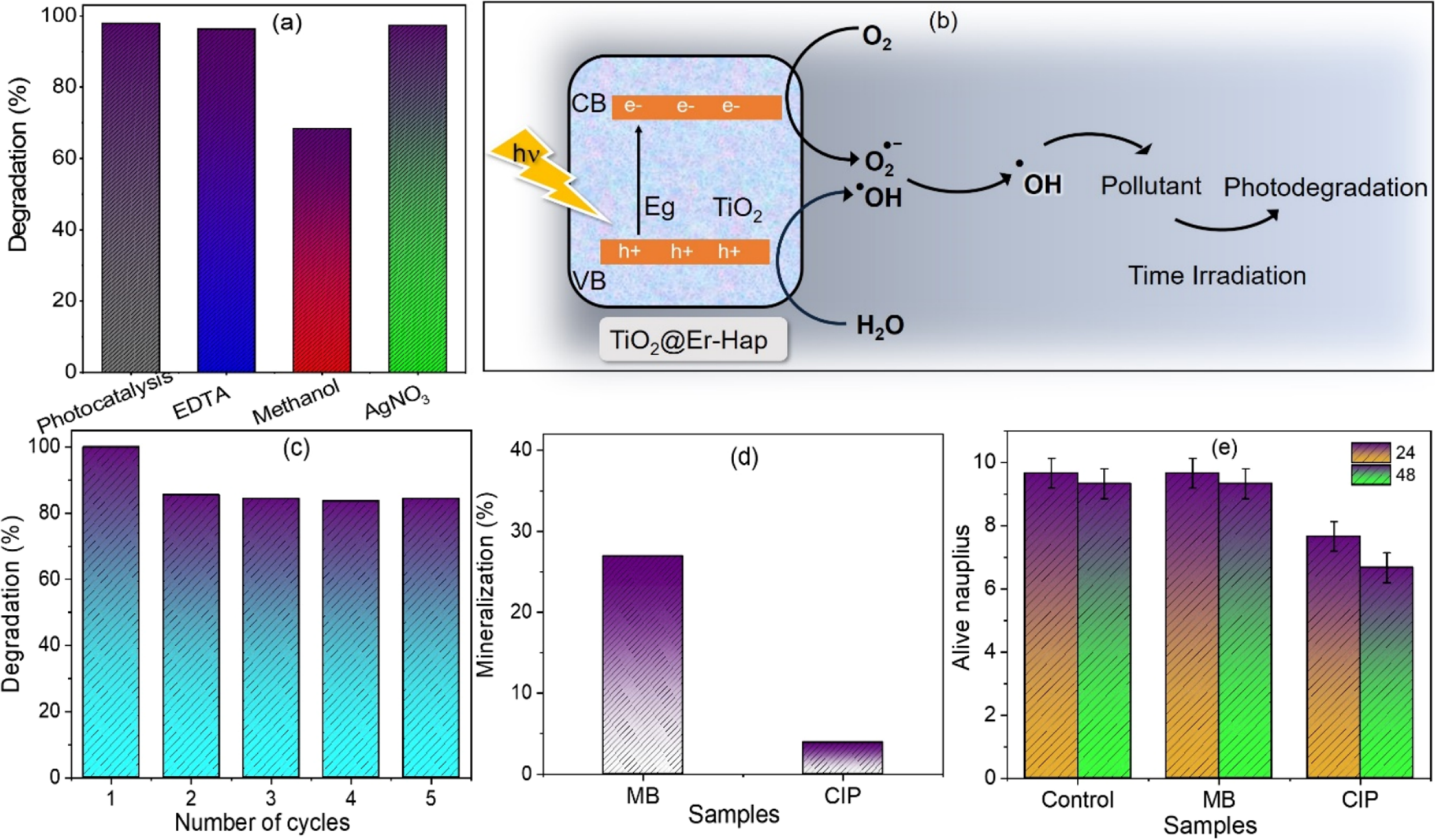
(a) Effect of scavengers on MB degradation in the presence of TiO_2_@Er-Hap, (b) photocatalytic mechanism, (c) reuse test, and (d) TOC analyses and (e) ecotoxicity test performed with *A. saline*.

As TiO_2_@Er-Hap was more efficient in removing the MB pollutant at all dosages tested, we performed inhibitor tests to determine which species were responsible for the photocatalytic performance observed for the material. The test was conducted by using the highest dosage of the catalyst, and the results are presented in Figure 7a. The addition of AgNO_3_ (e^–^ scavenger) and EDTA (h^+^ scavenger) in the photocatalytic process with the TiO_2_@Er-Hap material did not cause apparent changes in the discoloration of the MB. However, when methanol (^•^OH scavenger) was used, it was observed that there was a decrease in its photocatalytic efficiency, with a value of 68.38%. This result suggests the critical role of the ^•^OH species in the photocatalytic process.^6,55^ The ^•^OH radical is generally responsible for degrading organic pollutants and toxins in photocatalysis.^6^ These species are not selective and are associated with mineralizing organic pollutants. The photocatalytic activity in TiO_2_@Er-Hap is due to the oxide immobilized on the surface of the calcium phosphate. It is known that in TiO_2_ when it absorbs energy photons, the electron from the valence band is promoted to the conduction band of the semiconductor. In this case, an electron–hole pair is formed (eq 4). Once these charge carriers are generated, they migrate to the surface. Redox reactions are expected to promote the generation of ROS species, among which ^•^OH radicals (eqs 5–7) are important, which will degrade organic structures (eq 8). The reactions are described below, and a possible mechanism involved in the photocatalytic response by TiO_2_@Er-Hap is shown in Figure 7b.
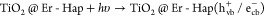
4
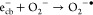
5
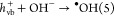
6
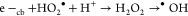
7
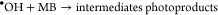
8

### Photocatalyst Recyclability

Using the highest dosage of TiO_2_@Er-Hap photocatalyst for MB degradation, a reuse test was carried out to investigate the stability of the incorporation of TiO_2_ in the material. These results are presented in Figure 7c, which considers five consecutive cycles. The first reuse cycle after the original photocatalysis demonstrated that the catalyst achieved a dye removal efficiency of 85.58%. Continuing with material recovery and reuse, it was observed that the MB removal capacity by TiO_2_@Er-Hap remained high even after the fifth consecutive cycle, with an MB degradation capacity of 84.37% observed. This result indicates that TiO_2_ remains stable on the surface of the erbium-doped hydroxyapatite. The slight loss in MB removal capacity may occur due to the accumulation of intermediate photoproducts blocking the active sites.^56^ However, TiO_2_@Er-Hap still showed satisfactory photocatalytic activity against MB dye, and this encourages us to classify this material as having the potential for removing pollutants from water bodies.

### Mineralization and Ecotoxicity Test

The mineralization of MB and CIP solutions irradiated with the most efficient dosage of TiO_2_@Er-Hap to degrade these pollutants was determined in this study, and the results are presented in Figure 7d. After 150 min of UV irradiation, the catalyst activated with UV irradiation achieved 26.90% for MB and 3.90% for the CIP drug. The percentage of mineralization promoted by TiO_2_@Er-Hap catalyst was higher for the dye when compared to the drug. The heterogeneous photocatalysis is based on the formation of oxygen radical species, especially ^•^OH radicals, which promote the degradation of organic structures into less complex molecular compounds.^57^ However, mineralization is not always achieved, and the formation of intermediate photoproducts is possible, as reported in the literature.^38^

The determination of photoproducts is challenging and requires more advanced techniques. However, it is possible to evaluate the toxicity of irradiated solutions using simple methods, e.g., based on plants and microorganisms.^58−60^ The bioassay using *A. saline* is simple, quick, low-cost, and well-accepted in the ecotoxicity assessment because these microcrustaceans are very sensitive to contaminants. The results presented in Figure 7e demonstrate that the number of *A. saline* nauplii that survived 24 and 48 h of contact with MB and CIP solutions after photocatalysis with TiO_2_@Er-Hap is greater than 50%. It indicates that the photoproducts that originated in photocatalytic systems are nontoxic for microcrustaceans.^41^ Regardless of the pollutant used, the solution treated after photocatalysis was nontoxic, indicating that the photocatalytic system worked.

## Conclusions

A novel crystalline material of Er-Hap decorated with TiO_2_ anatase was obtained successfully, as demonstrated in the XRD results. The oxide nanoparticles were agglomerated on the surface of calcium phosphate, and the presence of TiO_2_ resulted in a lower band gap energy and an increase in the surface area of the material. TiO_2_@Er-Hap was efficient in removing MB and CIP pollutants under UV irradiation. The decrease in the MB degradation rate in tests carried out with the material indicated that ^•^OH radicals were the main species involved in the photocatalytic activity promoted by TiO_2_@Er-Hap. This composite showed excellent stability because it could reuse the collected material up to five consecutive times. Therefore, the material synthesized shows promising properties for applications as a photocatalytic material for the degradation of water pollutants.

## Supporting Information Available

Supporting Information presents a plot of the PL emission spectrum for a TiO_2_@Er-Hap sample using an excitation source at 364 nm (PDF)
