# Supplementary material for: Enhanced Photocatalytic Performance of TiO2@Er-Hydroxyapatite Composite for Cationic Dye and Drug Removal
Source: ACS Omega. 2025 Feb 3;10(6):5351–61. doi: 10.1021/acsomega.4c06314 (PMC11840775; doi:10.1021/acsomega.4c06314)
Supplement: Supplementary file 1 — ao4c06314_si_001.pdf [file ao4c06314_si_001.pdf]

Enhanced Photocatalytic Performance of TiO_2@Er-Hydroxyapatite composite for Cationic Dye and Drug Removal

*Rafael Lisandro P. Rocha [a], Alan Ícaro S. Morais [a], Francisca P. Araujo [a], Luzia Maria C. Honório [b], Marcos P. Silva [a], Marcelo B. Furtini [a], Ewerton G. Vieira [c], Edson C. da Silva-Filho [a], Josy A. Osajima [a]**

[a] Interdisciplinary Advanced Materials Laboratory (LIMAV), Materials Science and Engineering Graduate Program, Federal University of Piaui (UFPI), 64049-550, Teresina, PI, Brazil.

[b] Department of Chemistry and Physics - Center for Agrarian Sciences, UFPB, Areia, PB, Brazil.

[c] Instituto Federal do Maranhão - Campus Buriticupu, IFMA, 65393-000, Buriticupu, MA, Brazil.

* Corresponding author: josyosajima@ufpi.edu.br

Supplementary material

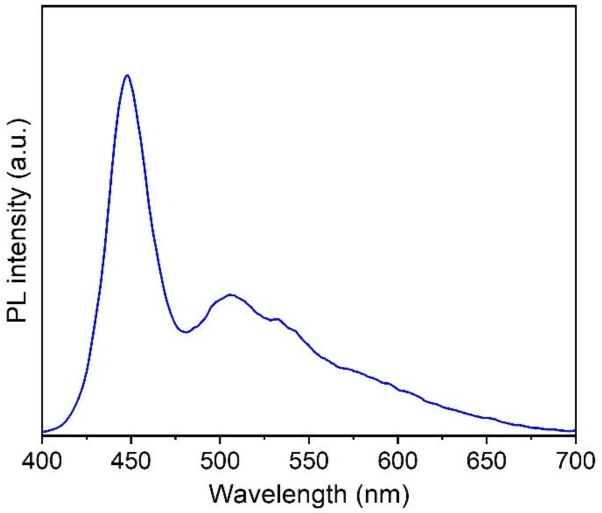

Figure S1. PL emission spectrum for TiO_2@Er-Hap sample using an excitation source at 364nm.
